# Supplementary material for: Nephrotoxicity of Immune Checkpoint Inhibitors in Mice with a Human Immune System
Source: bioRxiv. 2026 May 12:2026.05.07.723340. Preprint. [Version 1] doi: 10.64898/2026.05.07.723340 (PMC13192905; doi:10.64898/2026.05.07.723340)
Supplement: Supplement 1 [file media-1.pdf]

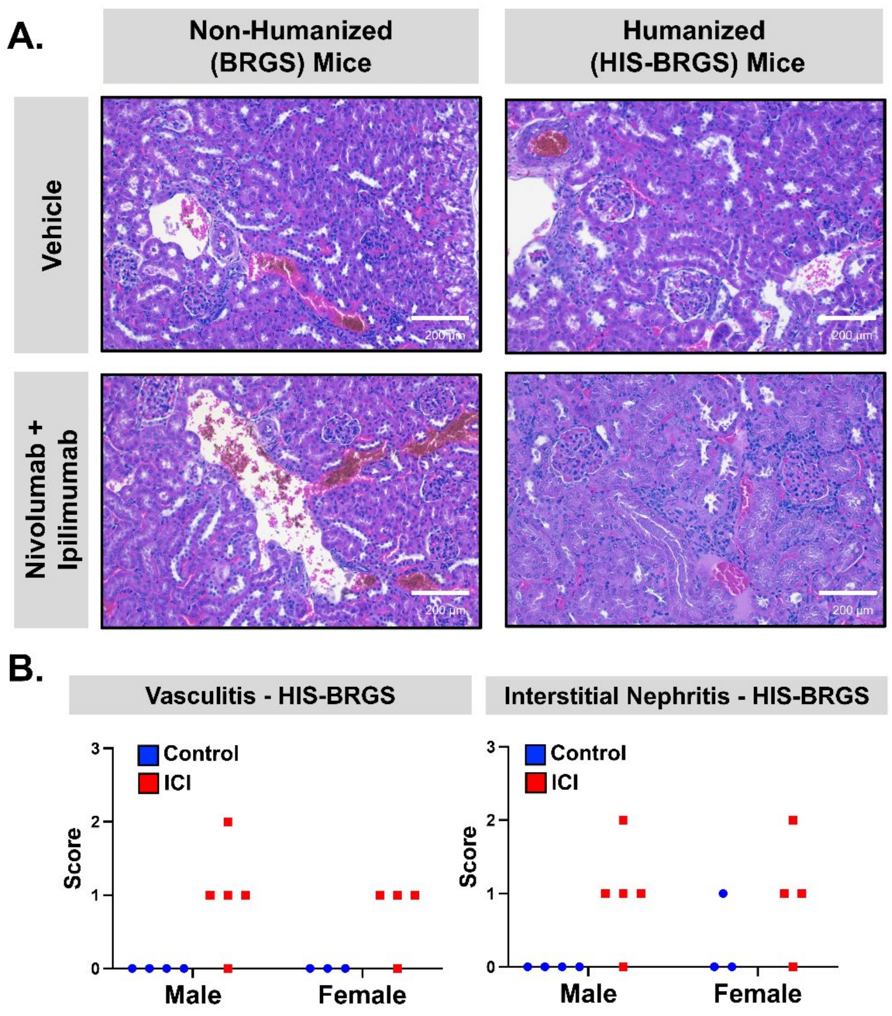

Supplemental Figure 1. Kidney Histopathology By Animal Sex. Humanized HIS-BRGS and non-humanized BRGS mice were implanted with tumors, followed by treatment with vehicle control (PBS) or immune checkpoint inhibitors (ICI, nivolumab and ipilimumab, 20 and 10 mg/kg weekly, i.p.). Kidneys were fixed in formalin and embedded in paraffin before sectioning and staining with H&E. (A) Light microscope images were acquired at 10X magnification. (B) Vasculitis and interstitial nephritis were assessed according to a range of scores (no lesions = 0; minimal lesions, <10% kidney affected = 1; mild lesions, 10-25% kidney affected = 2; moderate lesions, >25-40% kidney affected = 3) by a veterinary anatomic pathologist and displayed according to animal sex.

Supplemental Table 1. Flow Cytometry Antibodies and Reagents

Flow Cytometry Antibodies					Flow Cytometry Reagents	
**Target**	**Species**	**Fluorochrome**	**Clone**	**Source**	**Reagent**	**Source**
**CD45**	anti-human	BUV395	HI30	BD Bioscience	Zombie Green	BioLegend
**CD5**	anti-human	Fitc	UCHT2	BioLegend	L/D Blue	ThermoFisher
**CD3**	anti-human	PE	HIT3a	BioLegend	Rec Hu IL6	R&D Systems
**CD11b**	anti-human	PE	ICRF44	BioLegend	Rec Hu SCF	R&D Systems
**CD14**	anti-human	PE	63D3	BioLegend	Rec Hu FLT3L	R&D Systems
**CD33**	anti-human	PE	P67.6	BioLegend	FCS	Gibco
**PD-1**	anti-human	PE	EH12.2H7	BioLegend	FCS CD34+ media	Stemcell
**CD3**	anti-human	PeCy7	HIT3a	BioLegend	HBSS	Gibco
**CD45**	anti-human	BUV395	HI30	BD Bioscience	IMDM	Gibco
**CD45**	anti-human	PeCy7	HI30	BioLegend	Golgi Stop	BD Bioscience
**IFNγ**	anti-human	PeCy7	4S.B3	BioLegend	Cell Stim Cocktail	Invitrogen
**FoxP3**	anti-human	PacB	259D	BioLegend	Saponin	Sigma
**CD45**	anti-human	BV421	HI30	BioLegend	BSA (Bovine serum Albumin)	Sigma
**CD11c**	anti-human	BV421	s-hcl-3	BioLegend	Formaldehyde	Fisher
**HLA-DR**	anti-human	BV480	G48-6	BD Bioscience	Transcription Factor Staining Buffer Set	eBioscience
**Granzyme B**	anti-human	BV510	GB11	BioLegend		
**CD45RA**	anti-human	BV605	HI100	BioLegend		
**CD197/ CCR7**	anti-human	BV650	GO43H7	BioLegend		
**CD8**	anti-human	BV711	RPA-T8	BioLegend		
**CD4**	anti-human	BV785	RPA-T4	BioLegend		
**TIGIT**	anti-human	Kiravia Blue 520	A15153G	BioLegend		
**CD56**	anti-human	APC	HD56	BioLegend		
**CD19**	anti-human	APC	HIB19	BioLegend		
**TNFα**	anti-human	APC	MAb11	BioLegend		
**CD34**	anti-human	APC		Biolegend		
**CD25**	anti-human	Spark NIR685	M-A251	BioLegend		
**PD-1**	anti-human	AF700	EH12.2H7	BioLegend		
**CD8**	anti-human	APCFire	RPA-T8	BioLegend		
**mCD45**	anti-mouse	APCCY7	30-F11	BioLegend		
**CD19**	anti-human	APCFire810	HIB19	BioLegend		
**FCR Block**	human			Miltenyi		
**CD32**	mouse	BUV395	2.4G2	BioLegend		

Supplemental Table 2. Limits of Detection (LOD) for Analytes Used for Multiplex

Human Immune Response Panel					
**Analyte**	**LOD**	**Analyte**	**LOD**	**Analyte**	**LOD**
**April**	185	**CXCL-6**	0.7	**IL-15**	3.1
**BAFF**	2.3	**CXCL-8**	2.7	**IL-16**	15.7
**CCL1**	1.1	**CXCL-9**	10	**IL-17/CTLA-8**	3.8
**CCL2**	2.6	**CXCL-10**	2.2	**IL-18**	9.7
**CCL3**	3.1	**CXCL-11**	12.1	**IL-20**	13.9
**CCL4**	9.1	**CXCL-13**	15.2	**IL-21**	10.1
**CCL7**	4.4	**FGF-2**	10.2	**IL-22**	17.5
**CCL8**	1	**Galectin-3**	1007.6	**IL-23**	15.2
**CCL11**	2	**GM-CSF**	13.6	**Il-27**	10.3
**CCL13**	15.2	**Granzyme-A**	7.7	**IL-31**	16.1
**CCL17**	0.7	**Granzyme-B**	6.4	**IL-34**	10.7
**CCL19**	48.3	**HGF**	1.1	**IL-37**	2.2
**CCL20**	11.3	**IFN alpha**	8.9	**LIF**	4.6
**CCL21**	14	**IFN gamma**	10.7	**M-CSF**	16.1
**CCL22/MDC**	19.3	**IL-1 alpha**	1.9	**MIF**	0.9
**CCL23**	34.3	**IL-1 beta**	7	**MMP-1**	4.2
**CCL24**	5.4	**IL-2**	10	**NGF-β**	5.9
**CCL25**	10.2	**IL-2R**	100.4	**PTX-3**	29.4
**CCL26**	1.6	**IL-3**	31.8	**SCF**	4.5
**CD30**	8.5	**IL-4**	13	**TNF alpha**	14.6
**CD40L**	12.4	**IL-5**	10.2	**TNF beta**	0.7
**CSF-3**	14	**IL-6**	12.6	**TNF-RII**	2.9
**CX3CL1**	3.5	**IL-7**	1	**TRAIL**	3.4
**CXCL1**	2.8	**IL-9**	0.9	**TREM-1**	474
**CXCL2**	1	**IL-10**	4.2	**TSLP**	3.9
**CXCL5**	8	**IL-12p70**	7.2	**TWEAK**	137.7
		**IL-13**	4.7	**VEGF alpha**	6
**Mouse Kidney Injury Biomarker Panels**					
**Analyte**	**LOD**	**Analyte**	**LOD**	**Analyte**	**LOD**
**B2-Microglobulin**	50	**IP-10**	5	**Renin**	50
**Clusterin**	200	**KIM-1**	10	**TIMP-1**	20
**Cystatin C**	50	**NGAL**	5	**VEGF**	1
**EGF**	40	**OPN**	10		
**Human Immune-Oncology Checkpoint Protein Panel**					
**Analyte**	**LOD**	**Analyte**	**LOD**	**Analyte**	**LOD**
**CD80**	11.2	**CTLA-4**	9.3	**PD-L1**	1.3
**CD86**	86.1	**PD-1**	13.7		

Supplemental Table 3. Primers for qPCR Analysis of Human or mouse Genes.

Target	5' → 3'	3' → 5'
**Human**		
**AK2**	TCCTACCACGAGGAGTTCAACC	TGGTAGGCTTGCAGGCGGATTT
**ASXL2**	GGACAGAATCCAGGTGCGAAAAG	GATGGAGACTGGAAAACGAGCC
**CCS**	CAGAATGGAGGATGAGCAGCTG	GAGCGTGCAATGATGCCACAGG
**CCR5**	TCTCTTCTGGGCTCCCTACAAC	CCAAGAGTCTCTGTCACCTGCA
**CD226**	GGTGATACAGGTGGTTCAGTCAG	GGCTGGATCTTTTCCCACCTCA
**CD3D**	GCATCACATGGGTAGAGGGA	ACAGCTCTGGCACATTCGAT
**CD3E**	CGGTGGCCACAATTGTCATA	TTTCCGGATGGGCTCATAGT
**CD3G**	TCCTTGCTGTTGGGGTCTAC	GGAGAACACCTGGACTACTCTG
**CD5**	CGAGTTCTTGCCCTCCTTTGCT	TCCTGGCTGAAGAGCTGTCACA
**CD45**	TTCCTCGTCTGATAAGACAACAGT	TCTTTGCTGTAGTCAATCCAGTG
**CD84**	CTTCCAGACTCCTGAGGACCAA	ACATAGCCAGCACGCTCAGCAA
**CD8A**	TGCAACCACAGGAACCGA	TGCTCCCTCAAAAGGAAGGAT
**CD8B**	CCGGAAGACAGTGGCATCTA	TACAAAGTGGGCCCTTCTGG
**CXCL10**	AGTGGCATTCAAGGAGTACCT	TGATGGCCTTCGATTCTGGA
**CXCL13**	TATCCCTAGACGCTTCATTGATCG	CCATTCAGCTTGAGGGTCCACA
**GZMA**	CCACACGCGAAGGTGACCTTAA	CCTGCAACTTGGCACATGGTTC
**GZMB**	CGACAGTACCATTGAGTTGTGCG	TTCGTCCATAGGAGACAATGCCC
**GZMK**	TCCAGTATGGCGGACATCACGT	CGCCTAAAACCACAGTGGGAGA
**IL-15**	TTTGGGCTGTTTCAGTGCAG	ACTTTGCAACTGGGGTGAAC
**IL-16**	CAAGTCTCTCAAGGGGACCA	TGTGGCCTCTGCTGTAGATT
**ICOS**	CCCATAGGATGTGCAGCCTTTG	GGCTGTGTTCACTGCTCTCATG
**IL7R**	ATCGCAGCACTCACTGACCTGT	TCAGGCACTTTACCTCCACGAG
**LNPEP**	CAATGGCGGATAGAAAGCTGGTG	GCCATAGGTCATTCCACCACTTC
**OPTN**	ACTCTGACCAGCAGGCTTACCT	CTATGTCAGGCAGAACCTCTCC
**PGM2**	CGGATGCTGATAGACTTGCTGTG	TGAGAGCACTGCGATCCTGGTT
**PIK3R1**	CGCCTCTTCTTATCAAGCTCGTG	GAAGCTGTCGTAATTCTGCCAGG
**SAT2**	CTGAGAGCAGATGGCTTTGGAG	CCTCCAGATAAATGGTGCGTCC
**SHC1**	ACAGCCGAGTATGTCGCCTATG	CAATGGTGCTGATGACATCCTGG
**SLC12A4**	CTGTAACAGGCATCATGGCTGG	CACACTGCTGAAGTACACGAGG
**TAGAP**	ATGACTCCCTGGAGCACACTGA	CTGTTGGATTCCACATCAGGGTC
**Mouse**		
**Gapdh**	GTTCCTACCCCCAATGTGTC	GTTGAAGTCGCAGGAGACAA

Supplemental Table 4. mRNA Expression of Podocyte Essential Genes in Vehicle-Treated BRGS and HIS-BRGS Mice.[1]

	Veh BRGS		Veh HIS-BRGS		
**Target**	**Mean**	**SE**	**Mean**	**SE**	**P-adj**
**Aox1**	0.57	0.38	0.16	0.03	0.0792
**Cd59a**	9.24	0.41	6.32	0.35	0.0044
**Epb41l5**	8.50	0.32	6.34	0.20	0.0006
**Ezr**	131.49	7.27	101.20	2.74	0.0018
**Fnbp1l**	7.43	0.31	6.28	0.15	0.0532
**Golim4**	3.64	0.07	2.76	0.10	0.0052
**Ift80**	1.46	0.08	1.09	0.05	0.0195
**Itgav**	18.63	1.92	14.36	0.80	0.0774
**Itgb5**	32.59	1.41	40.12	1.55	0.0284
**Lpl**	64.01	5.65	24.55	1.53	0.0000
**Magi2**	1.41	0.08	1.09	0.06	0.0583
**Mtss1**	12.33	0.54	6.87	0.43	0.0000
**Myom2**	2.33	0.21	1.10	0.10	0.0000
**Nebl**	0.64	0.02	0.40	0.04	0.0166
**Nupr1**	3.47	0.46	5.29	0.82	0.1703
**Plce1**	1.33	0.02	0.91	0.07	0.0156
**Podxl**	54.62	3.86	36.67	2.86	0.0091
**Robo2**	1.21	0.12	0.73	0.09	0.0441
**Sema3g**	24.98	1.22	20.02	1.28	0.1682
**Shisa3**	4.26	0.52	2.10	0.18	0.0000
**Synpo**	8.85	0.15	5.55	0.34	0.0002
**Tdrd5**	0.89	0.07	0.65	0.04	0.0963
**Thsd7a**	2.09	0.13	1.05	0.08	0.0000
**Wt1**	2.01	0.09	1.13	0.09	0.0002

[1] Bulk RNA Sequencing was performed on total RNA from kidneys of vehicle-treated BRGS (n=3) and HIS-BRGS mice (n=7). Podocyte-essential genes were selected from Lu et al. and expressed as fragments per kilobase of transcript per million (FPKM) values and reported as mean and standard error (SE).

Supplemental Table 5. Circulating Human Cytokines, Growth Factors, Chemokines, Proteases, and Soluble Receptors in Vehicle- and ICI-Treated BRGS and HIS-BRGS Mice.[1]

	Veh BRGS		ICI BRGS		Veh HIS-BRGS		ICI HIS-BRGS	
**Target**	**Mean**	**SE**	**Mean**	**SE**	**Mean**	**SE**	**Mean**	**SE**
**BAFF**	1.65	0.00	1.65	0.00	43.10	13.24	94.69	19.42
**CCL1**	0.77	0.00	0.77	0.00	73.66	29.59	162.66	39.17
**CCL17**	0.48	0.00	0.48	0.00	10.03	5.24	24.94	6.40
**CCL20**	18.05	6.93	16.91	8.92	18.02	8.68	10.33	1.83
**CCL22/MDC**	13.65	0.00	13.65	0.00	67.93	34.10	97.97	26.54
**CCL24**	3.82	0.00	3.82	0.00	46.33	40.11	35.26	13.91
**CCL25**	4.41	1.64	5.86	1.35	7.21	1.59	20.60	4.35
**CCL7**	3.10	0.00	5.54	2.44	7.15	2.54	14.33	1.34
**CD30**	6.04	0.00	6.04	0.00	422.53	143.33	2666.99	880.30
**CSF-3**	10.82	0.92	9.90	0.00	14.59	4.11	18.52	4.13
**CXCL1**	3.17	0.82	2.32	0.21	14.58	4.12	14.61	2.93
**CXCL10**	1.58	0.00	1.58	0.00	97.60	74.01	146.82	53.07
**CXCL11**	8.56	0.00	8.56	0.00	19.47	7.07	32.18	12.95
**CXCL8**	15.02	12.17	12.82	3.72	78.25	36.48	125.61	43.39
**CXCL13**	10.72	0.00	10.72	0.00	2113.44	801.92	2625.73	515.09
**FGF-2**	146.23	91.32	69.44	21.21	195.53	119.74	223.70	25.58
**Galectin-3**	712.48	0.00	712.48	0.00	747.94	35.46	17306.94	9441.35
**GM-CSF**	9.62	0.00	9.62	0.00	41.78	32.16	145.74	76.28
**Granzyme-A**	5.44	0.00	5.44	0.00	7.29	3.20	33.20	20.94
**HGF**	0.60	0.17	0.72	0.18	0.83	0.30	1.70	0.40
**IL-2R**	70.99	0.00	70.99	0.00	895.26	697.36	4849.32	2415.56
**IL-3**	22.49	0.00	22.49	0.00	23.45	0.97	22.49	0.00
**IL-6**	8.89	0.00	8.89	0.00	154.80	115.64	213.72	118.85
**IL-15**	2.21	0.00	2.21	0.00	28.43	9.20	56.36	12.75
**IL-16**	11.10	0.00	11.10	0.00	61.26	31.03	381.74	149.74
**IL-18**	14.43	8.39	7.81	0.95	46.82	22.89	52.14	15.09
**IL-34**	10.37	2.83	7.54	0.00	8.98	2.53	21.23	4.44
**LIF**	224.43	30.92	203.46	23.65	118.87	20.24	218.16	13.82
**MIF**	37.14	7.91	24.59	3.41	18.75	4.74	36.21	6.29
**MMP-1**	8.05	5.64	12.22	3.87	78.38	30.53	102.95	60.49
**PTX-3**	349.27	316.47	65.19	25.47	54.50	9.90	88.67	37.78
**TNF-RII**	2.07	0.00	2.07	0.00	25.79	12.88	67.69	23.56

[1]Proteins below the LOD: APRIL, CCL-2, CCL-3, CCL-4, CCL-8, CCL-11, CCL-26, CCL-13, CCL-19, CCL-21, CCL-23, CD40L, CTLA-8, CXCL-2, CXCL-5, CXCL-6, CXCL-9, CX3CL1, Granzyme B, IFN alpha, IFN gamma, IL-1 alpha, IL-1 beta, IL-2, IL-3, IL-4, IL-5, IL-7, IL-9, IL-10, IL-12p70, IL-13, IL-20, IL-21, IL-22, IL-23, IL-27, IL-31, IL-37, M-CSF, NGF-β, SCF, TNF alpha, TNF beta, TRAIL, TREM1, TSLP, TWEAK, VEGF alpha

Supplemental Table 6. Human Cytokines, Growth Factors, Chemokines, Proteases, and Soluble Receptors in the Kidneys of Vehicle- and ICI-Treated BRGS and HIS-BRGS Mice.

	Veh BRGS		ICI BRGS		Veh HIS-BRGS		ICI HIS-BRGS	
**Target**	**Mean**	**SE**	**Mean**	**SE**	**Mean**	**SE**	**Mean**	**SE**
**April**	561.01	20.00	559.07	19.52	632.11	84.24	627.87	103.35
**BAFF**	3.25	0.08	3.26	0.07	6.23	0.55	11.22	0.95
**CCL1**	0.85	0.02	0.81	0.03	8.87	2.17	32.40	6.34
**CCL2**	0.74	0.09	0.67	0.06	4.65	2.09	2.51	0.75
**CCL3**	1.15	0.16	1.18	0.10	1.82	0.28	4.33	2.07
**CCL4**	4.96	0.61	4.93	0.35	6.27	0.56	12.24	3.94
**CCL7**	11.48	0.18	11.49	0.21	12.91	0.65	13.61	0.84
**CCL8**	0.34	0.04	0.35	0.02	0.43	0.04	0.37	0.03
**CCL11**	0.99	0.08	1.01	0.04	1.02	0.06	0.98	0.10
**CCL13**	16.22	1.63	16.22	0.84	27.33	5.94	18.90	2.16
**CCL17**	1.14	0.11	1.13	0.07	2.36	0.44	5.28	2.19
**CCL19**	124.50	8.65	129.30	6.33	152.87	15.32	167.99	36.03
**CCL20**	35.75	0.99	35.74	1.61	40.83	3.71	39.58	6.84
**CCL21**	31.61	1.44	33.75	1.42	47.85	7.62	49.76	12.22
**CCL22/MDC**	10.45	1.19	10.47	0.58	13.34	1.35	23.45	7.46
**CCL23**	22.47	0.94	22.24	0.66	38.56	4.43	36.99	5.92
**CCL24**	3.35	0.31	3.39	0.21	4.10	0.42	3.99	0.34
**CCL25**	8.54	0.92	9.09	0.68	10.69	1.05	11.07	1.42
**CCL26**	1.66	0.13	1.67	0.10	2.62	0.32	2.79	0.46
**CD30**	4.29	0.39	4.52	0.23	5.90	0.55	11.80	2.71
**CD40L**	10.62	0.77	10.99	0.70	17.71	2.38	17.81	3.55
**CSF-3**	70.82	11.73	70.30	6.70	77.38	10.63	69.22	8.18
**CX3CL1**	5.25	0.17	5.23	0.20	5.85	0.62	5.80	0.75
**CXCL1**	1.16	0.12	1.18	0.10	1.92	0.22	1.71	0.10
**CXCL2**	2.03	0.10	2.23	0.09	2.79	0.30	2.87	0.44
**CXCL5**	1.40	0.36	1.22	0.22	4.12	1.57	2.52	0.37
**CXCL6**	1.03	0.08	1.07	0.05	1.22	0.10	1.19	0.17
**CXCL8**	16.22	4.46	14.91	3.64	28.77	4.50	36.48	7.14
**CXCL-9**	58.19	2.49	61.92	2.10	69.14	7.11	71.38	11.34
**CXCL10**	1.15	0.11	1.18	0.13	7.63	4.35	19.52	8.00
**CXCL11**	30.69	0.31	30.72	1.14	34.71	4.05	35.40	5.30
**CXCL13**	34.04	1.81	33.31	1.22	115.98	24.66	415.58	222.81
**FGF-2**	80.62	11.82	70.77	6.43	70.60	4.26	68.91	9.82
**Galectin-3**	1663.95	76.13	1558.82	40.51	1759.88	151.28	1742.75	200.75
**GM-CSF**	15.34	2.19	14.83	1.17	18.31	1.33	18.58	2.08
**Granzyme-A**	2.73	0.23	2.95	0.17	9.33	2.00	49.68	16.62
**Granzyme-B**	5.86	0.40	5.84	0.17	7.23	0.87	18.26	7.46
**HGF**	0.69	0.02	0.67	0.03	1.03	0.16	1.05	0.16
**IFN alpha**	0.63	0.00	0.63	0.00	1.22	0.43	1.34	0.46
**IFN gamma**	1.06	0.32	1.21	0.20	1.42	0.20	2.12	0.49
**IL-1 alpha**	2.49	0.36	2.57	0.22	3.22	0.35	3.22	0.44
**IL-1 beta**	1.69	0.28	1.70	0.13	2.52	0.34	2.50	0.36

**IL-2**	3.25	0.42	3.37	0.23	3.52	0.24	3.12	0.39
**IL-2R**	63.75	4.74	54.80	10.08	123.09	19.33	181.68	55.80
**IL-3**	106.11	7.31	105.62	5.42	131.41	21.19	128.78	23.64
**IL-4**	7.08	2.02	10.99	1.16	22.39	3.91	11.61	2.63
**IL-5**	10.34	0.20	9.74	0.28	10.55	1.12	10.70	1.74
**IL-6**	8.66	1.40	7.82	0.79	23.34	8.47	30.28	12.03
**IL-7**	1.29	0.15	1.47	0.09	1.69	0.17	1.71	0.26
**IL-9**	0.76	0.06	0.81	0.05	0.83	0.07	0.83	0.10
**IL-10**	1.43	0.16	1.53	0.13	2.27	0.30	2.00	0.29
**IL-13**	1.16	0.03	0.94	0.11	1.13	0.17	1.14	0.14
**IL-15**	2.47	0.12	2.24	0.13	5.13	0.53	9.28	0.82
**IL-16**	7.56	1.14	7.80	0.67	12.36	2.22	123.08	63.37
**IL-17/CTLA-8**	2.20	0.24	2.24	0.14	2.61	0.24	2.73	0.26
**IL-18**	52.36	7.75	48.06	3.39	55.13	9.89	50.79	9.14
**IL-20**	9.14	1.14	9.71	0.60	10.05	0.80	9.65	0.90
**IL-21**	56.62	4.24	57.79	2.53	71.20	10.10	73.49	14.10
**IL-22**	43.43	1.32	43.99	2.01	53.70	8.73	52.65	10.72
**IL-23**	6.75	0.59	7.06	0.43	7.78	0.95	6.81	0.53
**IL-27**	1.77	0.62	1.99	0.46	5.71	0.19	4.51	1.65
**IL-31**	13.43	1.69	13.67	1.15	30.79	4.31	26.70	4.71
**IL-34**	9.72	1.24	9.08	0.64	13.47	2.51	13.79	2.73
**IL-37**	3.25	0.16	3.39	0.23	4.73	0.66	4.81	0.87
**LIF**	5.80	0.57	5.68	0.38	6.06	0.34	7.09	0.76
**M-CSF**	84.91	13.88	91.51	9.16	82.02	11.16	78.63	7.54
**MIF**	9.67	3.62	4.61	0.91	12.20	1.83	27.46	5.83
**MMP-1**	1.81	0.50	1.89	0.31	2.60	0.29	2.14	0.17
**NGF-β**	3.34	0.47	3.57	0.22	4.16	0.36	7.28	1.73
**PTX-3**	12.26	3.35	10.55	1.46	21.57	3.82	18.27	3.81
**SCF**	2.73	0.32	2.66	0.19	2.66	0.24	2.64	0.29
**TNF alpha**	0.67	0.13	0.65	0.12	1.65	0.34	1.84	0.38
**TNF beta**	0.49	0.02	0.45	0.03	0.61	0.09	0.61	0.12
**TNF-RII**	3.96	0.36	3.84	0.26	5.91	0.73	8.69	1.69
**TRAIL**	49.58	1.05	48.72	1.87	52.81	7.26	54.79	9.72
**TREM-1**	186.88	39.73	251.49	41.13	467.50	99.90	445.79	154.49
**TSLP**	8.50	0.91	8.89	0.63	14.06	1.80	14.04	2.45
**TWEAK**	66.92	10.57	55.76	4.89	80.81	4.71	92.67	8.00
**VEGF alpha**	19.55	4.39	24.32	4.08	25.64	1.77	22.17	3.50

[1]Proteins below the LOD: IL-12p70.
